# Bacterial Population Dynamics in a Laboratory Activated Sludge Reactor Monitored by Pyrosequencing of 16S rRNA

**DOI:** 10.1264/jsme2.ME12115

**Published:** 2012-10-26

**Authors:** Hiroyasu Satoh, Kenshiro Oshima, Wataru Suda, Purnika Ranasinghe, Ning Li, Egodaha Gedara Wasana Gunawardana, Masahira Hattori, Takashi Mino

**Affiliations:** 1 Department of Socio-cultural and Environmental Studies, Graduate School of Frontier Sciences, The University of Tokyo, 5–1–5 Kashiwanoha, Kashiwa, Chiba 277–8563, Japan; 2 Center for Omics and Bioinformatics, Graduate School of Frontier Sciences, The University of Tokyo, 5–1–5 Kashiwanoha, Kashiwa, Chiba 277–8561, Japan

**Keywords:** 16S ribosomal RNA, pyrosequencing, activated sludge, bacteriophage, T-RFLP

## Abstract

The microbial population in a laboratory activated sludge reactor was monitored for 245 d at 75 time points by pyrosequencing of 16S rRNA. Synthetic wastewater was used as the influent, and the reactor was operated under the same conditions throughout the experiment. The behaviors of different bacterial operational taxonomic units (OTUs) were observed. Multiple OTUs showed periodic propagation and recession. One of the OTUs showed sharp recession, which suggests that cells in the OTU were selectively killed. The behaviors of different phylogenetic lineages of *Candidatus* ‘Accumulibacter phosphatis’ were also visualized. It was clearly demonstrated that pyrosequencing with barcoded primers is a very effective tool to clarify the dynamics of the bacterial population in activated sludge.

Activated sludge processes are employed worldwide for the treatment of domestic and industrial wastewater. Pollutants in wastewater are removed by various types of microorganisms in the activated sludge. The bacterial population in activated sludge has attracted the interest of engineers and scientists because it affects the efficiency of wastewater treatment. For example, the control of filamentous microorganisms is necessary because they interfere with the gravimetric separation of biomass and treated water in settling tanks (14). Control of the competition between polyphosphate- and glycogen-accumulating organisms to allow the removal of phosphorus by the enhanced biological phosphorus removal (EBPR) method has been extensively investigated over the past two decades (15, 17, 21).

Various molecular methods are used for microbial population studies in activated sludge. The most commonly used methods are denaturing gradient gel electrophoresis (DGGE) (16), terminal-restriction fragment length polymorphism (T-RFLP) (13), cloning (9), real-time PCR, and fluorescent *in situ* hybridization (FISH) (1).

Electropherogram-based methods, such as DGGE and T-RFLP, enable the separation of different microbial groups as peaks in electropherograms, and the dynamics of each group can be determined by the change in peak intensity; however, the microbial species corresponding to the peaks of interest are usually identified by additional sequencing. A combination of cloning and sequencing or ribotyping can give information on phylogenetic identity; however, it is not an ideal method for quantitatively comparing microbial population structures in large numbers of samples. Real-time PCR and FISH give quantitative information on target microorganisms, although they do not provide insight into the whole microbial population structure.

Pyrosequencing overcomes the limitations of current methods. The 454 pyrosequencer (Roche, Basel, Switzerland) can perform around 10^6^ reads with a read length of around 400 bp in a single run and can analyze between 8 and 16 samples at a time. Using barcoded primers for PCR allows the source of the reads to be identified based on the barcode sequences; therefore, more samples can be analyzed simultaneously (2). Even when 100 samples are analyzed at a time, in theory around 10,000 reads can be obtained for each sample. The bacterial population structure can be constructed by using the phylogenetic identities of the reads. This approach is superior to conventional molecular methods because it provides both quantification and identification simultaneously.

In the present study, we introduced the pyrosequencing approach to observe bacterial population dynamics in activated sludge to demonstrate its efficiency. The monitored activated sludge process was a laboratory sequencing batch reactor, operated with anaerobic and aerobic sequencing to achieve enhanced biological phosphorus removal (EBPR). During its operation for 245 d, 75 samples were obtained and analyzed. We here report the dynamic behavior of bacterial population observed in the study. We cannot confirm that the reactor was operated under exactly the same operational conditions, in spite of our efforts; however, the dynamics found were much greater than expected, although it is not easy to explain our observations scientifically.

## Materials and Methods

### Laboratory sequencing batch activated sludge reactor

A laboratory sequencing batch reactor with a working volume of 10 L was used. The seed-activated sludge was obtained from a full-scale wastewater treatment plant that treats urban sewage. Each cycle consisted of the following sequence: influent synthetic wastewater (5 L) was fed in for 5 min, followed by a 55 min anaerobic phase, a 120 min aerobic phase, 45 min settling, and 15 min effluent discharge. Excess sludge was discharged during the aerobic phase. The nominal hydraulic retention time (HRT) was 8 h, and the nominal solids retention time (SRT) was 10 d. In practice, HRT and SRT were controlled mostly within ±20% of their target values. The sequence of anaerobic conditions with feed and aerobic conditions without feed promotes the selection of polyphosphate-accumulating organisms that contribute to the EBPR (15, 17, 21).

The nominal composition of the synthetic wastewater per liter of tap water was as follows: CH_3_COONa·3H_2_O (370 mg), CH_3_CH_2_COONa (130 mg), peptone (14 mg), yeast extract (17 mg), K_2_HPO_4_ (71 mg), CaCl_2_·2H_2_O (12 mg), MgSO_4_·7H_2_O (75 mg), KCl (30 mg), NH_4_Cl (75 mg), NaHCO_3_ (123 mg), allylthiourea (0.4 mg), FeCl_3_·6H_2_O (0.375 mg), H_3_BO_3_ (0.0375 mg), CuSO_4_·5H_2_O (0.0075 mg), KI (0.045 mg), MnCl_2_·4H_2_O (0.03 mg), Na_2_MoO_4_·2H_2_O (0.015 mg), ZnSO_4_·7H_2_O (0.03 mg), CoCl_2_·6H_2_O (0.0375 mg), and EDTA (2.5 mg). The substrate concentrations also have an error of ±20% because of the fluctuation of the flow rates of pumps. The reactor was operated in an air-conditioned room at 20±4°C. The reactor was run for 245 d, beginning on June 14, 2010.

Mixed liquor suspended solids (MLSS) and mixed liquor volatile suspended solids (MLVSS) concentrations in the aerobic phase, and phosphate concentrations and dissolved organic carbon (DOC) concentrations at the end of the anaerobic and aerobic phases were monitored at intervals ranging from 2 to 10 d. The concentrations of the MLSS and MLVSS were measured according to the standard methods (6). Phosphate concentrations were determined by the ascorbic acid method (6) or ion chromatography (IC-3000 and IonPac AS12A; Dionex, Sunnyvale, CA, USA), and DOC concentrations were determined using a TOC-VSCN TOC analyzer according to the instructions provided by the manufacturer (Shimadzu, Kyoto, Japan). Samples for bacterial analyses were during the aerobic period and stored at −80°C until use.

### Reverse-transcription PCR and pyrosequencing

The template for reverse-transcription PCR was prepared by the sonication-dilution method (21) with a minor modification. The frozen activated sludge samples were thawed and 1 mL of the thawed sample was sonicated (AD250; Branson, Danbury, CT, USA) at an amplitude of 30% (7 W) for 20 s. The sonicated samples were diluted 1,000-fold and were used as the template for reverse-transcription PCR.

The 27f and 519r primers (12) appended with 8-base barcode sequences were used (http://pyro.cme.msu.edu/pyro/help). The primer sequences used for each sample are listed in Table S1.

A PrimeScript One Step PCR Kit Version 2 (Takara, Otsu, Japan) was used for reverse-transcription PCR. The composition of the reaction mixture was as follows: 2× buffer (5 μL), forward primer (0.4 μL, 10 pmol μL^−1^), reverse primer (0.4 μL, 10 pmol μL^−1^), RNasin (0.1 μL; Promega), prime script enzyme mixture (0.2 μL), RNase-free water (2.6 μL), and the sonicated diluted activated sludge sample (1 μL). The thermal program was as follows: 50°C for 30 min, 94°C for 2 min, 20 cycles of 94°C for 30 s, 55.3°C for 30 s, 72°C for 30 s, and a final extension step of 72°C for 10 min.

The PCR product concentration was checked with a PicoGreen dsDNA Quantification Kit (Invitrogen, USA). The PCR products were purified with a High Pure PCR Clean Up Micro Kit (Roche). The quality of the purified PCR products was monitored with a 2100 Bioanalyzer (Agilent, Santa Clara, CA, USA).

The purified PCR products from samples taken on day 2 to day 112, and the samples taken on day 114 to day 245 were pooled, and the two pooled samples were subjected to standard pyrosequencing analysis using a Roche 454 FLX pyrosequencer.

### PCR and T-RFLP

A template for PCR was prepared by the same method used for reverse-transcription PCR. The primers used were 27f labeled with 6-carboxyfluorescein (FAM) at the 5′ end and 519r (13). The thermal program was as follows: 95°C for 10 min, 30 cycles of 94°C for 30 s, 55.3°C for 30 s, 72°C for 30 s, and a final extension step of 72°C for 10 min. The PCR product was purified with a QIAQuick PCR Purification kit (Qiagen, Valencia, CA, USA), digested with *Rsa*I (New England Biolabs, Ipswich, MA, USA), denatured, and separated using a 310 DNA sequencer (Applied Biosystems, Carlsbad, CA, USA).

### Sequence data analysis

The reads were assigned to their original samples with QIIME 1.5.0. The downstream primer region was manually removed with the text function of FileMaker Pro V10.0 (FileMaker, Santa Clara, CA, USA). Reads in the reverse direction were reverse-complemented and then combined with reads in the forward direction. The combined reads were classified into their operational taxonomic units (OTUs) by QIIME at 97% similarity. The phylogenetic affiliations were assigned by the Ribosomal Database Project classifier (25), aligned by the PyNAST algorithm, and the whole phylogenetic tree was calculated by the FastTree method using QIIME (4). A phylogenetic tree for selected OTUs was calculated by the neighbor-joining method using MEGA 5.0 (24). A partial phylogenetic tree was drawn using FigTree V1.3.1 (http://tree.bio.ed.ac.uk/). Our FileMakerPro Ver10 application, OTUMAMi (Operational Taxonomic Unit Management And Mining; available at http://www.mwm.k.u-tokyo.ac.jp:8080/Plone/), was used to select OTUs of interest and prepare heatmaps sorted by the partial tree order.

Virtual T-RFLP was performed for each sample with the reads obtained by pyrosequencing. For reads in which forward primer regions were found, the restriction site for *Rsa*I was identified, the expected sizes of the terminal-labeled restriction fragments (T-RFs) were calculated, and the number of reads with the same expected size was counted using OTUMAMi. For reads in which forward primer regions were found but restriction sites were not found, the sizes of the T-RFs were calculated only when reverse primer regions were found.

Statistical package R (20) was used for to obtain confidence intervals of polynominal distribution with package “binom” and the function “binom.lrt”.

### Nucleotide sequence accession numbers

The representative sequences of the major OTUs have been deposited in DDBJ with accession numbers AB736224 to AB736245.

## Results and Discussion

### Performance of the reactor

**Fig. 1:**
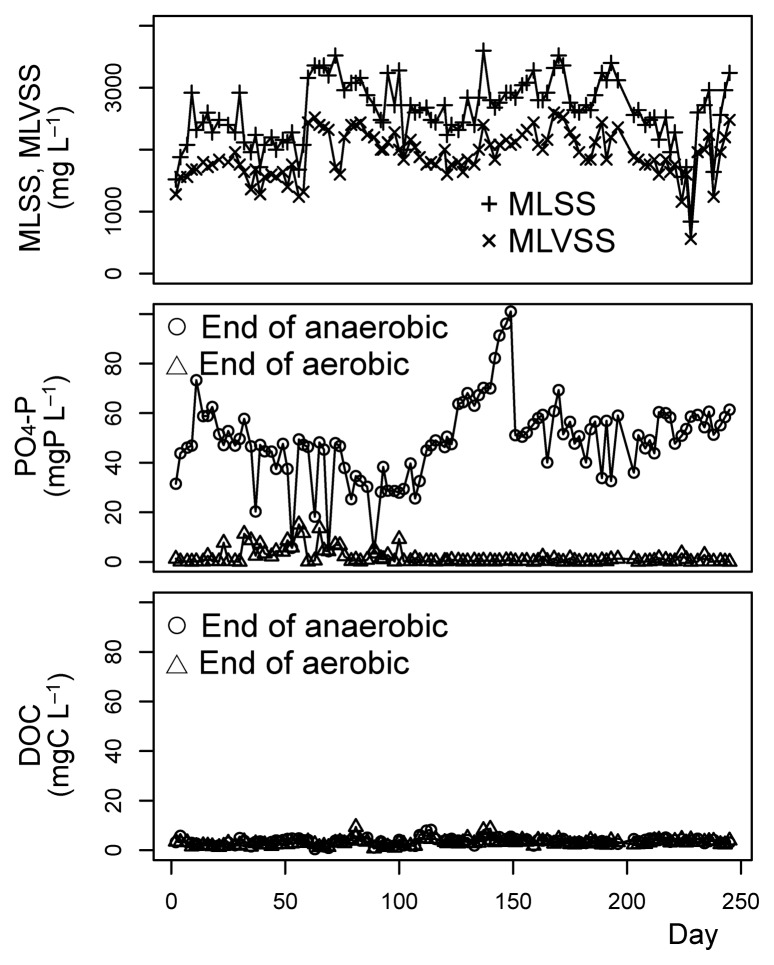
Performance of the laboratory sequencing batch reactor.

The performance of the reactor is shown in Fig. 1. The concentration of the MLSS was around 2,000 mg L^−1^ during the initial 60 d, and 3,000 mg L^−1^ from day 60 to day 200. After day 200, the MLSS gradually decreased to around 1,500 mg L^−1^, and then increased back to around 3,000 mg L^−1^.

Throughout the operation of the reactor, efficient EBPR was observed. The phosphate concentrations at the end of the aerobic phase were typically less than 0.5 mg-P L^−1^, whereas the concentrations at the end of the aerobic phase were usually higher than 30 mg-P L^−1^. The DOC concentrations at the end of the anaerobic phase were less than 5 mg-C L^−1^ whereas the influent DOC concentration was around 70 mg-C L^−1^. These observations are consistent with high polyphosphate-accumulating organism activity. Lower phosphate concentrations at the end of the anaerobic phase were occasionally observed from day 30 to 90, which was caused by the occasional failure of the influent. The causes of the sharp increase of biomass concentrations from day 58 to 60, the occasionally lower concentrations of MLSS and MLVSS after day 200, and higher phosphate concentrations at the end of the anaerobic phase during days 120 through 150 are not clear; however, we cannot eliminate the possibility that these situations were caused by technical problems that we failed to record.

### Bacterial population analysis

The number of reads obtained for the samples are listed in Table S1. In total, 86,440 reads were obtained. The minimum and maximum numbers of reads obtained for a sample were 246 and 8,217, respectively.

At thephylum level, 87.5% reads were affiliated with *Proteobacteria*, followed by *Bacteroidetes* (4.8%), *Verrucomicrobia* (1.9%), *Actinobacteria* (1.4%), and *Planctomycetes* (0.6%). At the class level, *Betaproteobacteria* accounted for 49% of total reads, followed by *Gammaproteobacteria* (24%), *Deltaproteobacteria* (12%), *Alphaproteobacteria* (3.1%), *Sphingobacteria* (2.9%), *Flavobacteria* (1.5%) and *Actinobacteria* (1.4%) (Fig. S1). Note that these genotypic compositions can be different from the real composition, because biases can be generated by the method of DNA extraction and by the choice of the primer set for PCR.

The whole reads, including the forward and reverse reads, were classified into 3,808 OTUs. For each sample, fractions *f_i,j_* and the mean fraction *f̄_j_* were calculated as follows:

(eq. 1) $$f_{i,j}=\frac{C_{i,j}}{\Sigma_{j}\;C_{i,j}}\times 100\;[\%]$$

(eq. 2) $${\bar{f}}_{j}=\Sigma_{i}\;f_{i,j}\times 100/N\;[\%]$$

where *C_i,j_* indicates the read count of OTU *j* in sample *i* and *N* is the number of samples.

Here, we focus on the 22 major OTUs with an *f̄_j_* bigger than 0.5%. The sum of *f̄_j_* for these OTUs was 60%, which means that three-fifths of the total reads were grouped into one of these major OTUs.

**Fig. 2:**
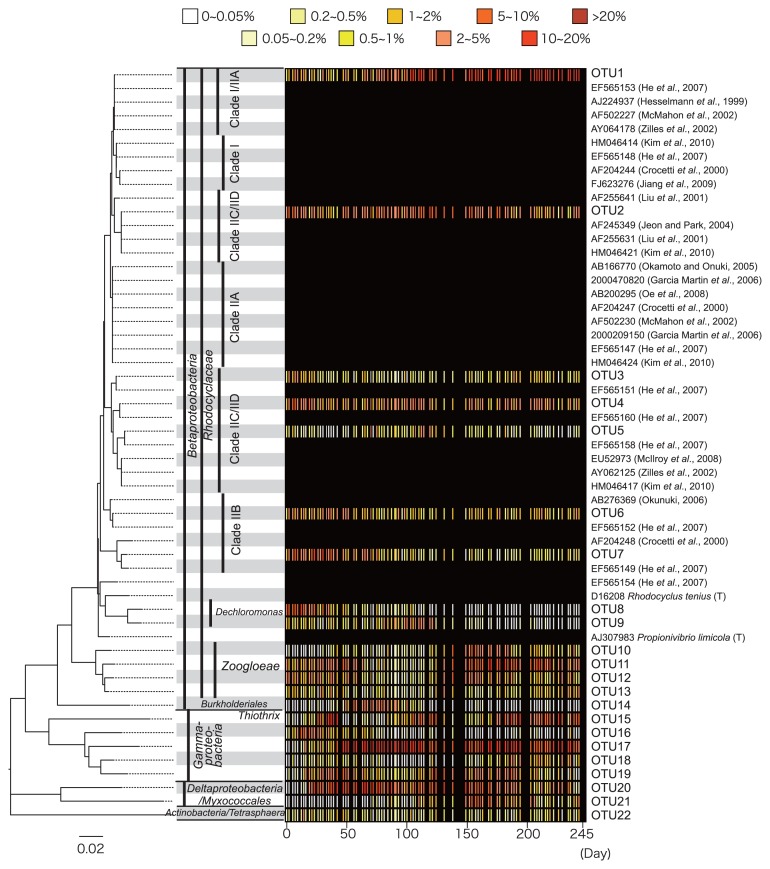
Heatmap of the major OTUs with reported *Candidatus* Accumulibacter-related sequences.

The combination of a phylogenetic tree and a heatmap for the major OTUs was constructed (Fig. 2). Fig. 2 also shows some type cultures within *Rhodocyclaceae* and the reported sequences for *Candidatus* ‘Accumulibacter phosphatis’, which is often the most important polyphosphate-accumulating bacteria responsible for EBPR. All the major OTUs belonged to the phylum *Proteobacteria*, except for one, which was classified into *Actinobacteria*. The Actinobacterial OTU was classified into *Tetrasphaera*, which is also reported to contribute to EBPR (11).

The propagation and recession of each OTU can be determined by using the heatmap. For example, OTU17 was one of the dominant OTUs in the reactor, particularly after day 50. During the latter half of the experiment, OTU1 was also dominant, whereas OTU8 was dominant during the first 20 d, and then almost disappeared.

Further, some of the OTUs showed repeated propagation and recession. OTU15 was dominant three times: from day 30 to day 50, from day 100 to day 130, and after day 180. Repeated propagation and recession was also observed for OTU2, OTU4, OTU5, OTU11, OTU18, and OTU19.

He *et al.* (8) classified *Candidatus* Accumulibacter by using the *ppk*I gene sequence and 1,000 bp of the 16S rRNA sequence, *E. coli* 16S rRNA gene positions 439 to 1,459. They classified the bacteria into 4 groups: Clade I, Clade IIA, Clade IIB, and Clade IIC/IID. As shown in Fig. 2, the resolution was not high enough to give perfect resolution of these groups: Clade I and IIA were found in the same branch (EF565153 and AF502227 are reported to be Clade IIA, while AJ224937 and AY064178 are Clade I), and Clades IIC/IID are separated into two branches. One possible cause for the difference is thought to be the size of the regions as only 500 bp was analyzed. The phylogenetic tree in Fig. 2 also shows that the major OTUs in *Rhodocyclaceae* could be classified into these clades, except for those which were classified into genus *Zoogloea*.

The OTUs in Clade I or Clade IIA were dominant, especially after day 100, and Clade IIB OTUs were also abundant until around day 50; therefore, the *Accumulibacter* population showed dynamic behavior even though the reactor operating conditions and the EBPR performance were similar throughout the experiment.

**Fig. 3:**
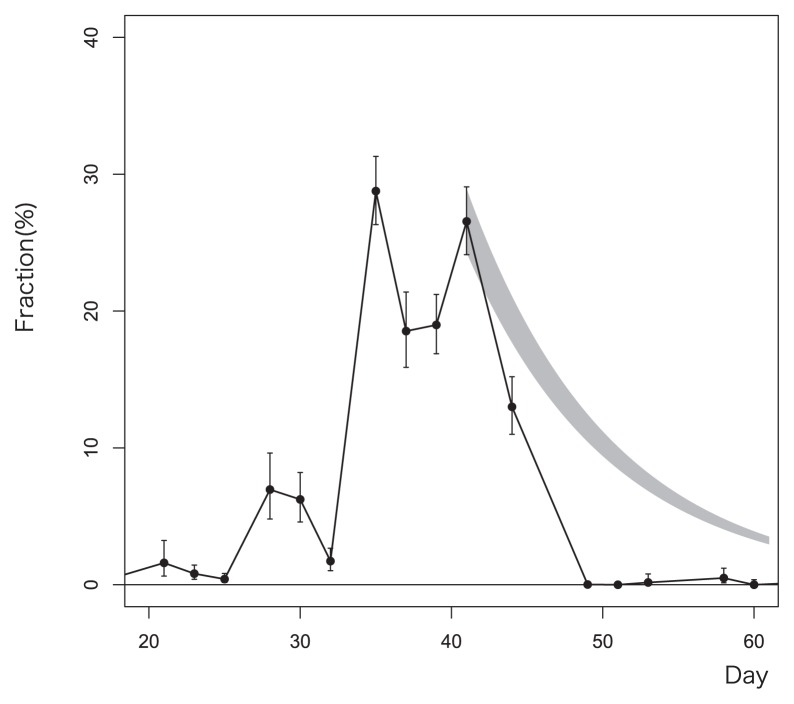
Dynamics of OTU15 for days 20 to 60. The shaded curve after day 41 is a calculated reduction curve for when the growth of OTU15 should have stopped. The bars for each plot represent confidence intervals of 95%.

As Fig. 2 shows, the *Betaproteobacteria* OTUs and OTUs in other classes exhibited dynamic behavior with repeated propagation and recession. The most impressive example is that of OTU15, which is shown in detail in Fig. 3. The expected error bars are calculated based on the polynomial distribution model. OTU15 dominated the whole population from day 30 to around day 45, and then almost disappeared. The shaded curve starting from day 41 in Fig. 3 shows the expected reduction curve calculated under the assumption that growth of OTU15 completely stopped and the population was then reduced by the withdrawal of excess sludge. The observed amounts of OTU15 were smaller than expected, which suggests that the OTU15 cells were selectively killed. Indeed, during days 45 and 49, only OTU15 reduced their abundance significantly, while others stayed almost the same and some rather increased their abundance. After day 50, OTU15 underwent propagation twice more, from days 100 to 125 and after day 190, and then underwent a recession between day 125 and day 175.

**Fig. 4:**
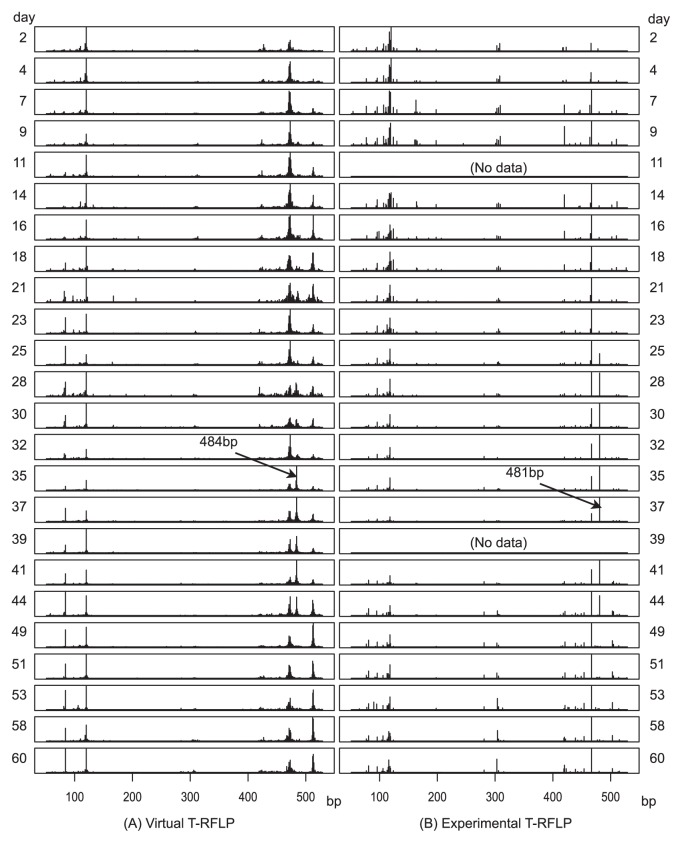
Comparison of the results of (A) the reverse-transcription PCR followed by pyrosequencing and virtual T-RFLP, and (B) PCR followed by T-RFLP. The experimental T-RFLP profiles are not the original electropherograms, and have been redrawn using the identified peak intensities.

Microbial behavior was examined by T-RFLP. Although pyrosequencing was conducted in combination with reverse-transcription PCR using a barcoded primer, T-RFLP was performed in combination with PCR without the barcode sequences. T-RFLP analysis was compared with virtual T-RFLP, which was calculated based on the pyrosequencing results (Fig. 4). The expected fragment length of OTU15 was 484 bp, and the corresponding 481 bp peak behaved as predicted, although for the experimental T-RFLP, the peak was more intense for day 25 to 32. While pyrosequencing was performed for reverse-transcription PCR products, T-RFLP was performed for products from PCR without reverse-transcription reaction; that is, experimental T-RFLP patterns reflected the abundance of the 16S rRNA gene, while virtual T-RFLP patterns reflected the abundance of 16S rRNA molecules. It is generally known that when a microbial species run into a growth phase, the number of ribosomes in a cell increases; the opposite was observed in the present study.

The cause of the sharp recession of OTU15 during day 45 and 49 is of interest. One possible cause is technical problems, but if technical problems caused the fluctuation of OTU15, how did they affect only selected OTUs, not all? Technical problems could have triggered the fluctuation, but it is rather difficult to explain the behavior of OTU15 solely by technical problems. As it appears as if OTU15 was “selectively killed”, it is more reasonable to look for possible mechanisms of selective killing. We consider that the possibility of grazing by protozoa or metazoa is rather low, because their feeding pattern is not thought to be so specific. Rather, the intervention of chemicals toxic or substances harmful only to OTU15 can be considered. A bacteriocin is a proteineous toxin that is selectively toxic to bacterial strains which are closely related to the bacteria that produce the bacteriocin (5). Regarding selectiveness, it is of interest that, in general, bacteriocin is thought to affect only closely related bacteria. In the case of OTU15, no closely related OTU was significantly detected. It is noticeable that OTU19 increased when OUT15 decreased, but they differed at the order level. Another possibility is lytic bacteriophages. They have high specificity to the host and can cause massive lysis of host bacterial cells within a short period of time, as is known for different phage-host systems. Further, the existence of bacteriophages hosted by bacteria in activated sludge has been reported previously (3, 19, 23).

The unusually rapid propagation of OTU15 from day 20 to 30 can also be seen in Fig. 2 and 3. OTU15 became dominant within a very short period of time at a much greater rate than any of the other OTUs in this study; that is, the fraction of OUT 15 was only 1.9% but it jumped to 29.9% on day 35. An increase at such a rate is possible only when more than half of the organic substrate has been utilized by OTU15, as nominal SRT was set to 10 d. Further work is required to discover what triggered and boosted their rapid growth.

Pyrosequencing was used to elucidate the behavior of bacteria in activated sludge at a superior taxonomic identification resolution, with satisfactory resolution in time and quantity. The bacterial population in activated sludge was not stable even when the operating conditions were constant; however, there was a short period when the influent feed failed and the cleaning of the reactors varied, both of which could have caused changes in the bacterial population. It is particularly difficult to explain the behavior of OTU15 by minor technical problems in the reactor operation. The results strongly suggest that factors inside the activated sludge, rather than the operating conditions, affected the bacterial population.

Changes in bacterial populations in laboratory activated sludge processes are often observed even when the processes are operated under the same conditions. Okunuki *et al.* (18) reported the deterioration and recovery of EBPR under the same conditions, suggesting that the microbial population changed for unknown reasons. Kaewpipat and Grady (10) and Gentile *et al.* (7) monitored microbial population changes in laboratory activated sludge reactors treating synthetic wastewater for 110 d by DGGE and for 325 d by T-RFLP. They concluded that the microbial population fluctuated dynamically because the peak patterns fluctuated, but did not report which species fluctuated or how they did so.

The PCR/pyrosequencing approach in combination with barcoded primers and bioinformatics analysis is a promising method for understanding the bacterial ecosystem in activated sludge. The fluctuation of different OTUs was successfully observed in a laboratory activated sludge reactor using this approach. Methods such as DGGE and T-RFLP can demonstrate dynamics, but it is difficult to prove that bands at the same position are really from the same species. The observation that some OTUs showed repeated propagation and recession can be confidently reported here because they are grouped based on sequences. Pyrosequencing makes it possible to grasp the dynamics of individual microbial species in activated sludge at a higher confidence of phylogenetic assignment than ever before.

As stated in the introduction, we cannot prove that the reactor was operated under exactly the same operational conditions, despite our efforts. Technical problems such as failure of feeding, failure of excess sludge removal, or overflow of mixed liquor were not rare, although not frequent. Thus, we hesitate to discuss the relationships between the bacterial population and treatment performance using the dataset reported here; however, we believe that the superior power of the pyrosequencing approach was very clearly demonstrated. Serious application of the technology to microbial population analyses in wastewater treatment processes will be the next step.

## Supplementary Material
