# Supplementary material for: Bacterial Population Dynamics in a Laboratory Activated Sludge Reactor Monitored by Pyrosequencing of 16S rRNA
Source: Microbes Environ. 2012 Oct 26;28(1):65–70. doi: 10.1264/jsme2.ME12115 (PMC4070689; doi:10.1264/jsme2.ME12115)

H. Satoh

Table S1 Primers used and read counts obtained for each sample.

		Group 1		Group 2	
Forward Primer	Reverse Primer	Sample(day)	Read Count	Sample(day)	Read Count
AGAGAGAG-27f	AGAGAGAG-519r	2	719	day114	114
AGAGATGC-27f	AGAGATGC-519r	4	932	day116	116
AGAGCAGC-27f	AGAGCAGC-519r	7	1459		
AGAGCATG-27f	AGAGCATG-519r	9	1212	day121	121
AGATCATC-27f	AGATCATC-519r	11	763	day123	123
AGATCTGC-27f	AGATCTGC-519r	14	924	day126	126
AGATGAGC-27f	AGATGAGC-519r	16	932	day133	133
AGATGATG-27f	AGATGATG-519r	18	404		
AGATGCAG-27f	AGATGCAG-519r	21	368	day140	140
AGATGCTC-27f	AGATGCTC-519r	23	1164		
AGCAGAGC-27f	AGCAGAGC-519r	25	1513		
AGCAGATG-27f	AGCAGATG-519r	28	428	day151	151
AGCAGCAG-27f	AGCAGCAG-519r	30	688	day154	154
AGCAGCTC-27f	AGCAGCTC-519r	32	980	day156	156
AGCATCTG-27f	AGCATCTG-519r	35	1263	day159	159
AGCATGAG-27f	AGCATGAG-519r	37	765	day161	161
AGCTCAGC-27f	AGCTCAGC-519r	39	1256	day163	163
AGCTCATG-27f	AGCTCATG-519r	41	1218	day165	165
AGCTGATC-27f	AGCTGATC-519r	44	983		
AGCTGCTG-27f	AGCTGCTG-519r			day170	170
ATCAGATC-27f	ATCAGATC-519r	49	5181	day172	172
ATCAGCTG-27f	ATCAGCTG-519r	51	906		
ATCATCAG-27f	ATCATCAG-519r	53	517	day177	177
ATCATCTC-27f	ATCATCTC-519r			day179	179
ATCTCATC-27f	ATCTCATC-519r	58	687		
ATCTCTGC-27f	ATCTCTGC-519r	60	523	day184	184
ATCTGAGC-27f	ATCTGAGC-519r			day186	186
ATCTGATG-27f	ATCTGATG-519r	65	315	day189	189
ATCTGCAG-27f	ATCTGCAG-519r	67	926	day191	191
ATCTGCTC-27f	ATCTGCTC-519r	69	742	day193	193
ATGAGAGC-27f	ATGAGAGC-519r	72	680	day196	196
ATGAGATG-27f	ATGAGATG-519r	74	420		
ATGAGCAG-27f	ATGAGCAG-519r	77	1336	day205	205
ATGAGCTC-27f	ATGAGCTC-519r	79	2131	day208	208
ATGATCTG-27f	ATGATCTG-519r	81	1544	day210	210
ATGATGAG-27f	ATGATGAG-519r	83	687	day212	212
ATGCAGAG-27f	ATGCAGAG-519r	86	1463	day214	214
ATGCATGC-27f	ATGCATGC-519r	89	1350	day217	217
ATGCTCAG-27f	ATGCTCAG-519r	92	1123	day219	219
ATGCTCTC-27f	ATGCTCTC-519r	93	932	day221	221
CAGAGAGC-27f	CAGAGAGC-519r	95	882	day224	224
CAGAGATG-27f	CAGAGATG-519r	98	740	day228	228
CAGAGCAG-27f	CAGAGCAG-519r	100	1297	day231	231
CAGAGCTC-27f	CAGAGCTC-519r	102	518	day236	236
CAGATCTG-27f	CAGATCTG-519r	105	905	day238	238
CAGATGAG-27f	CAGATGAG-519r	107	537	day241	241
CAGCAGAG-27f	CAGCAGAG-519r	109	518	day243	243
CAGCATGC-27f	CAGCATGC-519r	112	681	day245	245
				Total Count	52923

H. Satoh

Fig. S1. Taxonomic identities of the reads. The circles represent phylum (inside), class, order, family, and genus levels (outside). Gray part indicate close taxa were not assigned.

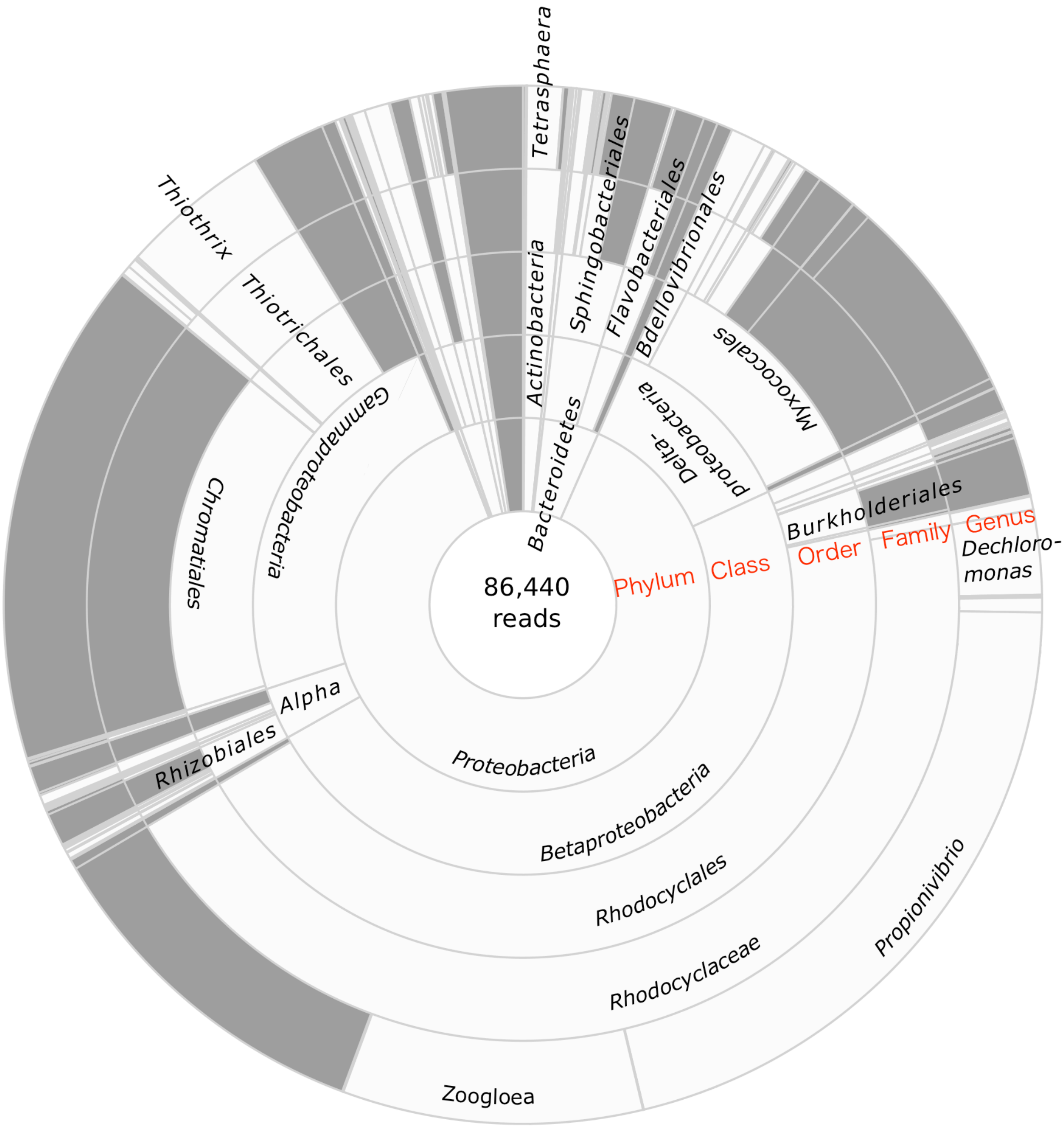

Supplement: Supplementary file 1 [file 28_65_s1.pdf]
